# Longitudinal Wastewater-Based Epidemiology Reveals the Spatiotemporal Dynamics and Genotype Diversity of Diarrheal Viruses in Urban Guangdong, China

**DOI:** 10.3390/v18010083

**Published:** 2026-01-08

**Authors:** Shuling Li, Jiadian Cao, Yuxi Yan, Wenwen Deng, Yuwei He, Siling Xiang, Chuting Zeng, Heshi Long, Shuxian Li, Qiao Yao, Biao Zeng, Baisheng Li, Song Tang, Jing Lu

**Affiliations:** 1 School of Public Health, Guangdong Pharmaceutical University, Guangzhou 510006, China; shulingli0412@163.com; 2 Guangdong Provincial Institution of Public Health, Guangdong Provincial Center for Disease Control and Prevention, Guangzhou 511430, China; caojd6@mail2.sysu.edu.cn (J.C.); yanyuxi0214@163.com (Y.Y.); dwwwendy@163.com (W.D.); hyw1284765894@163.com (Y.H.); xsl433789@163.com (S.X.); sun0_ft@163.com (C.Z.); 15777307630@163.com (H.L.); 3 Guangdong Provincial Key Laboratory of Pathogen Detection for Emerging Infectious Disease Response, Guangdong Workstation for Emerging Infectious Disease Control and Prevention, Guangdong Provincial Center for Disease Control and Prevention, Guangzhou 511430, China; stevenzeng202305@163.com (B.Z.); libsn@126.com (B.L.); 4 School of Public Health, Sun Yat-sen University, Guangzhou 510080, China; 5 School of Basic Medicine and Public Health, Jinan University, Guangzhou 510632, China; 6 School of Public Health, Southern Medical University, Guangzhou 510515, China; 7 College of Science, Shantou University, Shantou 515063, China; 8 School of Mathematics and Computational Science, Guilin University of Electronic Technology, Guilin 541004, China; 9 National Key Laboratory of Intelligent Tracking and Forecasting for Infectious Diseases, National Institute of Environmental Health, Chinese Center for Disease Control and Prevention, Beijing 100021, China; maxine_lee_lsx@163.com (S.L.); yaoqiao223@163.com (Q.Y.); 10 China CDC Key Laboratory of Environment and Population Health, National Institute of Environmental Health, Chinese Center for Disease Control and Prevention, Beijing 100021, China

**Keywords:** wastewater-based epidemiology, pathogen surveillance, diarrheic viruses, Norovirus, Astrovirus

## Abstract

Following the normalization of the COVID-19 pandemic, the focus of wastewater-based epidemiology (WBE) must be broadened from SARS-CoV-2 to encompass surveillance of other major infectious diseases, particularly for pathogens where conventional clinical monitoring systems exhibit inherent surveillance gaps. In this study, we conducted a continuous two-year WBE study (January 2023 to December 2024) across three high-population-density cities in Guangdong, China to establish epidemiological baselines for enteric diarrheal viruses. We analyzed monthly raw wastewater samples from major treatment plants using advanced molecular methods, including digital PCR (ddPCR) for viral load quantification and targeted high-throughput sequencing (tNGS) for genotypic analysis. Our findings revealed diverse circulation patterns among the monitored enteric viruses. Astrovirus (AstV) had the highest detection rate (100%), reflecting its broad endemic distribution, while Norovirus genogroup II (NoV GII) exhibited relatively high viral loads (median 4 × 10^4^ copies/mL) and presented explosive seasonal peaks (significant upward trend in spring.), highlighting its epidemic potential. Furthermore, distinct spatiotemporal patterns were observed, with Sapovirus showing a significant summer peak in Foshan city, contrasting with the winter/spring peaks in the other cities. The tNGS results demonstrated similar sensitivity to RT-PCR in virus detection, and sequencing analyses uncovered the co-circulation and periodic shifts in dominant viral genotypes, such as the emergence of multiple NoV and AstV lineages. This longitudinal WBE surveillance successfully established critical baseline data and demonstrated significant regional heterogeneity in viral circulation, providing essential, complementary data to inform public health strategies for preventing diarrheal outbreaks in urban settings.

## 1. Introduction

Diarrheal diseases represent a significant challenge in global public health, particularly in low and middle-income countries, contributing to a substantial number of child deaths annually [1]. Viruses are major pathogens causing foodborne outbreaks, and the proportion of virus-related cases and fatalities continues to rise [2,3]. Viral diarrhea, especially caused by Rotavirus A, Norovirus, Enteric Adenovirus, and Astrovirus, is among the most common etiologies of diarrhea in children under five years old [4]. Most countries and regions currently lack systematic surveillance systems for diarrheal pathogens. Instead, surveillance is often limited to outbreak-based networks, such as the US Centers for Disease Control and Prevention’s National Outbreak Reporting System (NORS), and CaliciNet in Europe, both of which mainly focus on monitoring norovirus outbreaks [5,6]. The diarrheal viruses are shed in large quantities in feces, with asymptomatic individuals potentially shedding up to 10^5^ to 10^12^ viral particles per gram of feces [7]. Therefore, wastewater surveillance offers a powerful, non-invasive approach to monitor the prevalence of diarrhea-related viruses in a population.

In recent years, wastewater-based epidemiology (WBE) has gained widespread attention as an innovative public health surveillance tool [8,9]. The WBE approach involves analyzing pathogen nucleic acids or metabolites present in urban wastewater systems, enabling a non-invasive and systematic reflection of the overall health status of the covered population [10,11]. Compared to traditional clinical surveillance methods, WBE offers several significant advantages, including its ability to capture the collective impact of both symptomatic and asymptomatic individuals on the community’s pathogen ecology. Its results are not influenced by individual healthcare-seeking behaviors, and it demonstrates high timeliness and cost-effectiveness [12,13,14]. While initially used for drug abuse monitoring, WBE rapidly expanded into viral surveillance during the COVID-19 pandemic, unequivocally proving its critical utility [15,16,17]. Despite WBE’s immense success in SARS-CoV-2 monitoring, systematic research on its application to other significant pathogens, particularly diarrheic viruses with complex prevalence patterns, remains relatively limited [18,19,20,21]. Moreover, wastewater serves as a critical reservoir for antibiotic resistance genes (ARGs), making it a powerful tool for monitoring antimicrobial resistance (AMR) [22]. Although integrated surveillance of viruses and ARGs is an ideal public health strategy, the viral component is often overlooked. Therefore, establishing a reliable baseline for viral pathogens is a fundamental step.

This study aims to bridge this critical scientific gap through a two-year, multi-center investigation that systematically explores the diarrheal virus circulation pattern in wastewater. Specifically, the research addresses the following core scientific questions: (1) Which diarrheal viruses can be consistently detected in wastewater, and possess significant monitoring value? (2) What are the baseline viral loads and distribution characteristics of these pathogens? (3) Do spatial differences and temporal trends exist in the distribution and prevalence of diarrheal viruses across different cities? (4) Do the viral genotypes and dominant strains change dynamically over time, particularly with the seasons?

Through the analysis of 2 years’ wastewater samples collected from three densely populated cities in Guangdong Province, this study provides the real-time wastewater-based surveillance on diarrheal virus which may reflect the viral load dynamics, spatiotemporal trends, and genotypic diversity of various diarrheal viruses within urban communities.

## 2. Materials and Methods

### 2.1. Study Area, Sample Collection, and Transportation

This study selected the influent of representative municipal wastewater treatment plants in Dongguan (DG), Foshan (FS), and Zhuhai (ZH) cities in Guangdong Province as sampling points [23]. Sampling occurred monthly from January 2023 to December 2024, spanning 24 months to obtain long-term time-series data. After collection, all samples were stored in 4 °C and transported to the laboratory of Guangdong Provincial Center for Diseases Control and Prevention (GDCDC) within 24 h for subsequent processing [24].

### 2.2. Virus Concentration and Nucleic Acid Extraction

Wastewater samples received at the laboratory underwent pre-treatment, starting with centrifugation of 20 mL of sample at 2000 g for 2 min at 4 °C to remove solid particles [25]. Subsequently, 17.5 mL of the supernatant was subjected to viral concentration using a viral particle magnetic bead enrichment method [26], yielding 60 µL of nucleic acid product. The extracted nucleic acid product was then used for subsequent real-time quantitative PCR (RT-qPCR), digital PCR (ddPCR), and target high-throughput sequencing (tNGS) analyses.

### 2.3. Quantitative Fluorescence Methods

This study employed fluorescence quantitative PCR (RT-qPCR) for the detection of nucleic acids from eight (Rotavirus A, Rotavirus B, Norovirus GI, Norovirus GII, Astrovirus, Enteric Adenovirus and Enterovirus) diarrheic viruses (KH25058, Sansure Biotech, Changsha, China) and digital PCR (ddPCR) (DQ24 Plus Digital PCR System, Suzhou Sniper Medical Technologies, Suzhou, China) for absolute quantification. According to the manufacturer, the quantitative PCR kit has a reported typical limit of detection (LOD) of approximately 500 copies/mL for each target. The magnetic bead-based viral enrichment method was validated pre-study using PMMoV, yielding a recovery of 34.4–53.6%. An internal control (IC) co-processed with each sample confirmed PCR inhibition (Ct SD < 0.5; CV < 2%). Standard curves were established by combining standards with specific primers and probes, thereby converting qPCR results into logarithmic values of viral copies per milliliter of sample (log_10_ copies/mL) [27].

### 2.4. High-Throughput Metagenomic Sequencing

We utilized the MetaVirus hybridization capture reagents and accompanying kits (KS668-MetaV-12) from KingCreate, Guangzhou, China for sequencing library preparation. First, viral nucleic acids were reverse transcribed and then fragmented into 200–500 bp segments, which were ligated with barcode-indexed adapters to prepare sample nucleic acid sub-libraries. Subsequently, target viral nucleic acid fragments were enriched by hybridization capture using a massive probe panel covering over 1300 viruses and more than 15,000 viral strains. Finally, the captured libraries were subjected to high-throughput sequencing to obtain raw sequencing data.

### 2.5. Sequencing Data Analysis

Raw fastq sequences were processed using fastp [28] to quality trim (Q25) and remove Illumina adapters. Processed reads were run through the EsViriru pipeline (v0.1.1), which was customized by incorporating more comprehensive set of norovirus reference genomes [29]. This read-mapping approach assigned dual genotypes based on the capsid region (VP1 for enteroviruses, ORF2 for noroviruses),allowing for the accurate identification of known recombinant strains. Virus genomes/segments with reads covering at least either 1000 nucleotides or 50% of the genome/segment length were considered preliminary detections. To correct for genome length bias, the relative abundance of each viral genotype was calculated as the proportion of its Reads Per Kilobase (RPK) value relative to the total RPK sum of all genotypes within the sample. Furthermore, negative, blank, and positive control samples were included in the experimental workflow, and measures such as aseptic operations, paired-end sequencing, and bioinformatics filtering were adopted to minimize exogenous contamination and ensure that sequencing data originated from authentic samples.

### 2.6. Data Quality Control and Statistical Analysis

During the long-term surveillance, there were a data gap (specifically in February 2023 for Zhuhai). We employed linear interpolation and extrapolation to complete the time series, ensuring the statistical power of trend analysis [30,31]. To validate the rationality of this imputation method, we performed Kolmogorov–Smirnov (KS) tests [32] on the measured values and imputed data for each virus in each city. The results showed that, except for a very few groups, the *p*-values of the KS test for most pathogen-city combinations were greater than 0.05, indicating no statistically significant difference in the distribution between the imputed and measured data. This powerfully demonstrates that this imputation method does not introduce significant bias into subsequent core conclusions regarding viral prevalence trends, seasonal characteristics, etc., providing a solid foundation for complete data series analysis.

### 2.7. Data Availability

All sequencing reads mapped to the diarrheal viruses have been deposited to the GSA database of National Genomics Data Center (https://bigd.big.ac.cn/, accessed on 8 October 2025) with submission number PRJCA046343.

## 3. Results

### 3.1. Overall Prevalence and Viral Load Distribution of Diarrheal Viruses

**Figure 1:**
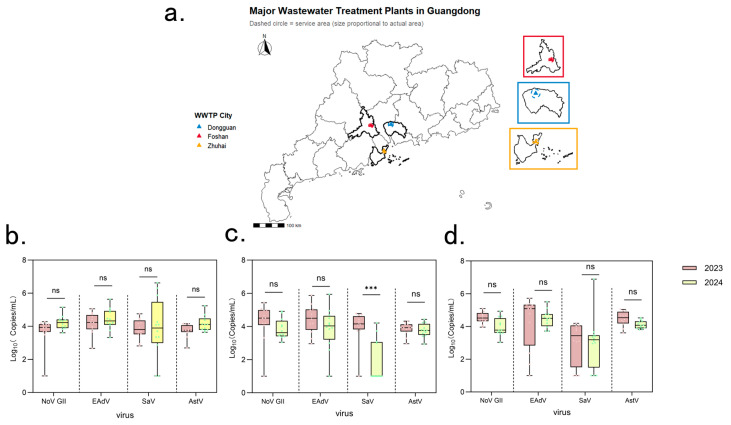
**Geographical Locations of Wastewater Treatment Plants (WWTPs) and Inter-annual Viral Load Comparisons (2023 vs. 2024) in Guangdong Province, China.** (**a**) Location of wastewater treatment plants (WWTPs) and drainage areas in Guangdong Province, China. This map illustrates the geographical location of the three sampled cities within Guangdong Province, China, along with the approximate boundaries of their respective wastewater drainage areas and the locations of the contributing wastewater treatment plants (WWTPs). (**b**–**d**) Comparative analysis of major diarrheal virus loads in influent wastewater from three cities between 2023 and 2024. (**b**) Foshan (**c**) Dongguan (**d**) Zhuhai: Boxplots show the concentrations (log_10_ copies/mL) of four major diarrheal viruses—Norovirus GII (NoV GII), Enteric Adenovirus (EAdV), Sapovirus (SaV), and Astrovirus (AstV)—in influent wastewater samples collected over two periods: 2023 (pink) and 2024 (yellow-green). The black horizontal lines represent median values, boxes indicate interquartile ranges (IQR), and whiskers show 1.5× IQR. Statistical differences between years were assessed by Student’s *t*-test; ns indicates no significant difference, and *** denotes *p* < 0.001. The results demonstrate temporal and spatial variations in viral loads across the three cities.

Guangdong is one of the regions in China with the highest population size and density. Given its distinct climate and critical role as a global trade and transport hub, the number of reported gastroenteritis outbreaks consistently ranks among the nation’s highest. To provide fundamental data on the detection rates of diarrhea-related viruses in regional wastewater, the RT-PCR testing was performed on 71 wastewater samples collected from three high population density cities (Foshan, Dongguan, and Zhuhai) in Guangdong, China (Figure 1a) over a two-year period. Astrovirus (AstV) exhibited the highest detection rate (100% in three cities), followed by enteric adenovirus (EAdV) (95.65–100%) and Norovirus GII (NoV GII) (95.83–100%). Norovirus GI (NoV GI) and Sapovirus (SaV) also showed relatively high detection rates, at 80.28% and 78.87%, respectively. In contrast, Enterovirus (EV) and Rotavirus A (RVA) had comparatively lower detection rates, at 39.44% and 11.27%, respectively, while Rotavirus B remained undetectable throughout the surveillance period. These data established a baseline for diarrheic virus detection rates in high-density urban areas of Guangdong Province, highlighting astrovirus and Norovirus GII were almost consistently presenting pathogens.

### 3.2. Viral Load and Epidemiological Characteristics

The digital PCR results provided crucial insights into the distribution and variability of viral loads across different pathogens and cities. Norovirus GII consistently exhibits a high median viral load (around 4 log_10_ copies/mL) and a wide interquartile range with numerous upward-extending outliers (Figure 1), reflecting significant variability and recurrent surges in viral load. This pattern is characteristic of highly contagious pathogens capable of causing explosive outbreaks with substantial epidemic risk. Conversely, Astrovirus, despite having a comparable median viral load, displays a much narrower and more concentrated distribution within its boxplots, with a smaller interquartile range and fewer outliers (Figure 1). This indicates a more stable and persistent shedding pattern across the population, reflecting a widespread and ubiquitous presence rather than the intense, high-load peaks observed in Norovirus GII. Enteric Adenovirus and Sapovirus generally show intermediate median viral loads and varying degrees of distribution width, suggesting more complex shedding patterns influenced by seasonal factors or localized transmission. During the spring peak (February to April), the median viral load of norovirus GII in three cities reached 44,033 copies/mL, which showed a significant upward trend in spring.

**Figure 2:**
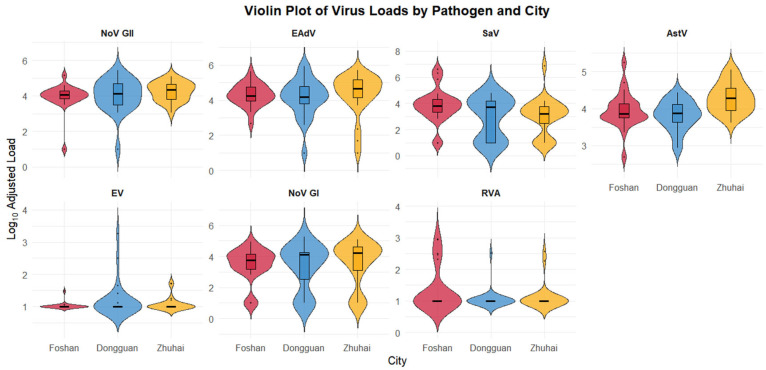
**Violin Plots of Adjusted Virus Loads for Seven Enteric Viruses across Foshan, Dongguan, and Zhuhai.** Violin plots illustrate the distribution of Log10 adjusted virus loads for seven different enteric viruses: Norovirus GII (NoV GII), Enteric Adenovirus (EAdV), Sapovirus (SaV), Astrovirus (AstV), Enterovirus (EV), Norovirus GI (NoV GI), and Rotavirus A (RVA). Each panel represents a specific virus, and within each panel, the red, blue, and yellow violin shapes correspond to data from Foshan, Dongguan, and Zhuhai, respectively. The width of each violin indicates the density of data points at different load values, with the embedded box plot showing the median (horizontal line), interquartile range (box), and potential outliers (dots). The figure highlights variations in both the central tendency and spread of virus loads among different pathogens and geographical locations, providing a comprehensive visual summary of viral load distributions.

The density distribution of viral loads across cities and viruses effectively delineated their characteristic shedding patterns and dynamic ranges (Figure 2). For Norovirus GII, the wide violin shapes with pronounced “tails” and higher densities at elevated loads corroborate its capacity for explosive, high-intensity shedding episodes. In contrast, Astrovirus’s narrower, more symmetrical violin shapes visually reinforce its consistent and widespread presence with less extreme variability, despite its high median load. Other viruses, such as Norovirus GI and enteric adenovirus, exhibited intermediate viral loads with varying violin widths. Rotavirus A and Enterovirus consistently showed low median loads (less than 2 log_10_ copies/mL) and narrow, symmetrical violin shapes, possible indicative of less intense, more sporadic detection.

**Figure 3:**
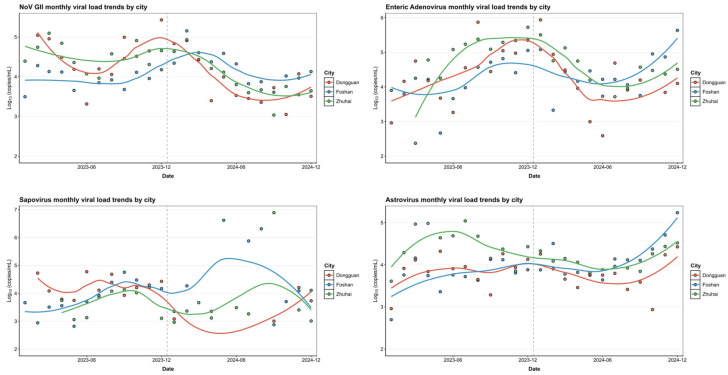
**Monthly temporal trends of the four predominant enteric viruses in wastewater samples across three cities.** Line plots display the monthly variations in viral concentrations (log_10_ copies/mL) for the four viruses with the highest loads: Norovirus GII (**top left**), Enteric Adenovirus (**top right**), Sapovirus (**bottom left**), and Astrovirus (**bottom right**). Data points represent individual sample measurements, and the fitted lines indicate smoothed trends for each city (Foshan, Dongguan, and Zhuhai). The results demonstrate that these viruses consistently exhibited the highest concentrations among all monitored pathogens, with distinctive temporal and spatial fluctuation patterns among the three cities.

Figure 3 illustrate how viral loads of different enteric viruses changed over time and across three cities (DG, FS, ZH) from 2023 to 2024. Astrovirus (AstV) and Enteric Adenovirus (EAdV) exhibited relatively stable viral loads over time in all three cities, suggesting a consistent and widespread presence in the population. Sapovirus (SaV) also generally maintained a high-level presence across all cities and years. However, a particularly noteworthy finding from the inter-annual comparison highlighted a highly significant difference (*p* < 0.001, indicated by ‘*’ and confirmed through multiple comparisons) in Sapovirus viral loads between 2023 and 2024 specifically in Dongguan (Figure 1c and Figure 3), indicating important localized year-to-year variation despite its overall high prevalence. Norovirus GII, in contrast, showed significant fluctuations in viral load across the months, with clear peaks and valleys (Figure 3). This pattern indicates a strong seasonal influence and a transmission mode characterized by explosive, epidemic-prone outbreaks. Enterovirus (EV) were generally present at low levels but had distinct, intermittent peaks (Figure 1 and Supplementary Figure S1a), suggesting a pattern of low-level circulation with occasional outbreaks. Norovirus GI also presented more variability and seasonality than AstV or EAdV (Supplementary Figure S1b), exhibiting distinct monthly variations across cities and suggesting a complex epidemiology. Contrary to the highly fluctuating patterns observed for Norovirus GII, Rotavirus A (RVA) generally maintained consistently low viral loads and detection rates throughout the study period, with limited temporal variation (Figure 1, Supplementary Figures S1c and S2a).

In sum, these findings reveal diverse epidemiological profiles among enteric viruses in wastewater in Guangdong. While some, like AstV and EAdV, show perennial presence, others exhibit clear seasonal patterns (e.g., NoV GII, NoV GI) or possible sporadic increases in viral load (e.g., EV), with specific viruses like SaV demonstrating nuanced year-to-year changes in particular locations.

### 3.3. Target Next Generation Sequencing Insights into Viral Genotypes and Temporal Dynamics

Target metagenomic sequencing complemented and validated the results obtained from RT-PCR, further elucidating the genotypic composition and dynamic changes within the community’s viral populations [33]. To gain deeper insights into the molecular epidemiology behind the observed viral load dynamics, we conducted a detailed case study using targeted metagenomic sequencing. A custom probe enrichment panell—targeting 38 human viruses, including all diarrheal viruses detected in this study—was applied to 12 wastewater samples collected specifically from Foshan during its 2024 peak season.

**Figure 4:**
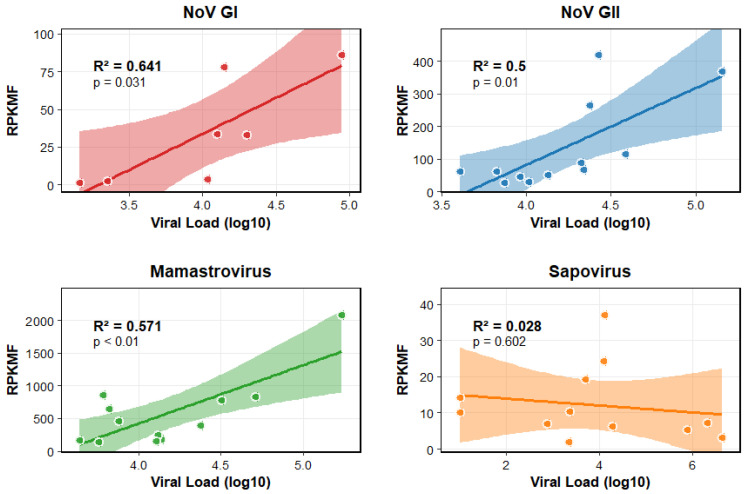
**Relationship Between Viral Load and RPKMF for Different Virus Types.** This figure illustrates the linear relationship between log10-transformed viral load and RPKMF (Reads Per Kilobase Million) for four virus types: NoV GI, NoV GII, Mamastrovirus, and Sapovirus. Each panel shows individual data points (colored by virus type), a linear regression line, and its 95% confidence interval (shaded area).

The detection sensitivity and quantitative agreement between the molecular methods were thoroughly assessed (Figure 4). Based on the established tNGS criteria (requiring a minimum of 10 reads per virus for positive detection), the overall detection rate of tNGS in samples that were positive by RT-PCR was high, ranging from 40% to 100% across different viruses. Conversely, the detection rate of target viruses by tNGS in RT-PCR negative samples was minimal (14.3–100%), indicating high specificity and consistency between the two platforms. A quantitative comparison revealed that the tNGS sequence abundance did not completely correlated well with the viral load obtained by ddPCR (Figure 4). Specifically, highly abundant viruses like Norovirus GI showed a relatively strong linear correlation (R^2^ = 0.64, *p* = 0.031) between the number of sequencing reads and the viral concentration. However, this linearity was less pronounced for viruses with lower loads, such as Rotavirus A (RVA), as well as Sapovirus. This diminished correlation is likely attributable to a combination of factors: low viral concentration approaching the limit of detection for tNGS, and potentially incomplete probe coverage for the high genetic diversity of certain viruses, which would restrict the proportion of target sequences captured during enrichment.

**Figure 5:**
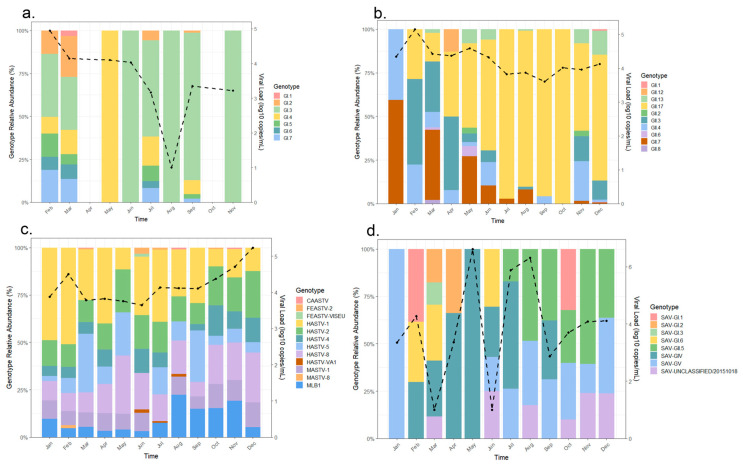
**Temporal dynamics of viral genotypes and viral load.** (**a**) Relative abundance of NoV GI genotypes, (**b**) relative abundance of NoV GII, (**c**) relative abundance of Astrovirus genotypes and (**d**) relative abundance of Sapovirus genotypes over time. The stacked bars show the proportion (left *y*-axis) of different genotypes (indicated by color). The black line tracks the corresponding viral load (right *y*-axis, log_10_ copies/mL). The results reveal shifts in genotype composition and viral load throughout the sampling period.

Sequencing results further refined the prevalence characteristics at the genotypic level (Figure 5). Among Noroviruses, genogroup II predominated, with GII.17 being the most abundant (20.3%), followed by GII.4 (18.5%) and GII.3 (14.8%). Norovirus GI was primarily composed of GI.3 (25.0%) and GI.4 (17.8%). Astrovirus was most frequently HAstV-1 (27.0%). Among Rotaviruses, G9P was the major type, accounting for 44.4%. These findings provide detailed genotypic “fingerprints” for viral diarrhea in the region, offering valuable references for public health interventions. Distinct dominant genotypes at various time periods, potentially influenced by environmental factors, host immune pressure, or other ecological conditions. Genotypic changes may also be linked to environmental and seasonal variations, with certain genotypes potentially gaining an advantage in specific seasons or conditions. Specifically, GI genotype distribution is relatively even, but during the high prevalence period in spring, genotypes such as GI.1 and GI.3 show increased proportions, which is also reflected in a clear peak in the viral load curve. This indicates that Norovirus GI has a strong prevalence momentum in spring. The dynamic changes among different genotypes of GI reflect relatively stable diversity. The performance of GI.3 and GI.7 suggests that these two types may be associated with specific transmission routes or host adaptations at different times. GII.4 and GII.17 predominated in spring (February to June), especially during viral load peaks. The proportional changes in these genotypes closely synchronized with viral load fluctuations, further supporting those specific types (such as GII.17) appears to have driven outbreaks. While other GII genotypes fluctuated during the same period, the dominant types were very prominent. HASTV-1 and HASTV-2 were the main dominant types, dominating in months with high total read percentages (e.g., December), with viral loads also increasing in autumn and winter, showing a possible positive correlation. This indicates that the high prevalence period of Astrovirus may be driven by certain genotypes, and the proportions between genotypes maintain a certain dynamic. The Sapovirus genotypic distribution showed high diversity and significant fluctuation, with no obvious monthly dominant type, though viral loads briefly increased in some months (e.g., August–September). Rotavirus A, however, had G9P [4] as the absolute dominant type, with genotype proportions remaining largely stable, consistent with its overall lower read percentages and viral load trends. Further longitudinal surveillance is warranted to fully elucidate the complex interplay between ecological factors, viral genotype distribution, and community viral load shedding patterns.

## 4. Discussion

This longitudinal WBE study across high-population density cities in Guangdong successfully established epidemiological baselines for diarrheal viruses and delineated two distinct transmission patterns.

WBE effectively differentiates the shedding dynamics of highly prevalent viruses. Astrovirus (AstV), with 100% detection and consistently high viral loads, reflects a persistent endemic shedding pattern, strongly suggesting widespread asymptomatic or mild infections within the community [34,35,36]. Conversely, Norovirus GII (NoV GII), exhibiting significantly higher loads and explosive spring peaks (consistent with prior regional surveillance similar with the pattern observed in our previous regional surveillance 2008–2019 [37]. Its violin plots show wide distributions with pronounced upward “tails” and higher densities at elevated load values, visually representing significant variability and recurrent surges (Figure 2). This pattern of high load and significant fluctuation is a typical indicator of acute, high-intensity transmission, forewarning a high risk of Norovirus GII causing community diarrheal outbreaks [38]. The distinct viral prevalence patterns through WBE provides public health authorities with a more nuanced risk assessment—identifying which viruses are persistently present versus which serve as potential outbreak warning signals.

Analysis revealed a complex pattern characterized by ‘macro-synchrony and micro-asynchrony’ across the three cities. From a macroscopic perspective, macro-synchrony (e.g., shared NoV GII spring peaks and predominant genotypes like GII.17) is attributed to the shared subtropical climate and the region’s status as the core of the Guangdong-Hong Kong-Macao Greater Bay Area (GBA). The macro-synchronous pattern was especially apparent during the emergence of the GII.17 genotype in the winter of 2014–2015 [39]. Our case study in Foshan provides a potential explanation for this phenomenon: the spring peak in that city coincided with the predominance of the GII.17 genotype. Given the geographical proximity and high interconnectivity, we hypothesize that similar genotypic shifts may have driven the concurrent peaks in Dongguan and Zhuhai. However, this remains a hypothesis that requires direct molecular surveillance in those cities for validation. This regional synchrony was less pronounced in the sporadic dynamics of Enterovirus (EV) and Norovirus GI (Supplementary Figure S1), suggesting that community-level outbreaks are also substantially influenced by independent local factors.

The application of Next-Generation Sequencing (NGS) in wastewater virome surveillance remains challenging. Firstly, the complex matrix of wastewater, coupled with low viral loads and severe nucleic acid fragmentation due to degradation, makes conventional metagenomic sequencing and PCR amplicon sequencing difficult to implement effectively. We explored the application of targeted Next-Generation Sequencing (tNGS) via probe enrichment to overcome the challenges of low viral load and nucleic acid fragmentation inherent in wastewater. The method demonstrated high sensitivity (comparable to RT-PCR) and successfully provided critical genotypic abundance data. The synergy between genotypic and quantitative data is evident. For instance, the spring peak coincided with the emergence of GII.17, suggesting a potential link between genotype shifts and seasonal dynamics (Figure 5). This integration highlights the potential for WBE to not only track transmission intensity but also to elucidate the predominant viral strains during outbreaks (e.g., NoV GII.17, AstV HAstV-1). Such molecular insights could be valuable for informing vaccine development, evaluating targeted interventions, and optimizing public health responses.

Despite its translational success, the study has certain limitations. A primary limitation is the lack of normalization for viral load data. The reported concentrations are ‘apparent concentrations’ and were not adjusted for wastewater flow rates or fecal strength markers like Pepper Mild Mottle Virus (PMMoV). Consequently, observed fluctuations, such as the spring peaks of Norovirus GII, may be influenced by hydraulic factors like dilution from rainfall, not solely by biological transmission dynamics. Future research must incorporate such normalization to accurately quantify community viral loads. Secondly, the genotype analysis was confined to Foshan in 2024, making any extrapolation of these specific viral patterns to other cities or time periods speculative without further targeted sequencing. Thirdly, the monthly sampling is insufficient for capturing short-term dynamics. The observed patterns should therefore be viewed as broad seasonal trends, and future work requires higher-frequency sampling for more precise epidemiological tracking. Finally, several technical factors inherent to wastewater surveillance must be considered. Maintaining comprehensive probe coverage across highly variable viruses remains a challenge for tNGS, as indicated by the disparity between RT-PCR positive EV samples and undetectable EV sequences. For samples with high Ct values (>35), the potential for non-specific signals, although minimized by stringent quality controls, cannot be completely ruled out. We also acknowledge that the accuracy of viral quantification can be affected by the inherent complexities of wastewater systems, including flow rate variations, dilution effects, and environmental inhibitors [40,41].

## Data Availability

All sequencing reads mapped to the diarrheal viruses have been deposited to the GSA database of National Genomics Data Center (https://bigd.big.ac.cn/, accessed on 8 October 2025) with submission number PRJCA046343.

## Supplementary Materials

The following supporting information can be downloaded at https://www.mdpi.com/article/10.3390/v18010083/s1, Supplementary Figure S1: Monthly temporal trends of Enterovirus, Norovirus GI, and Sapovirus viral loads in wastewater samples across three cities. (a) Enterovirus, (b) NoV GI, and (c) Sapovirus; Supplementary Figure S2: Temporal comparison of Rotavirus A, Enterovirus, and Norovirus GI viral loads in wastewater samples from three cities in Guangdong, China (2023 vs. 2024). (a) Foshan, (b) Dongguan, and (c) Zhuaihai.
