# Supplementary material for: Longitudinal Wastewater-Based Epidemiology Reveals the Spatiotemporal Dynamics and Genotype Diversity of Diarrheal Viruses in Urban Guangdong, China
Source: Viruses. 2026 Jan 8;18(1):83. doi: 10.3390/v18010083 (PMC12846444; doi:10.3390/v18010083)
Supplement: Supplementary file 1 [file viruses-18-00083-s001.zip › viruses-4047999-supplementary.pdf]

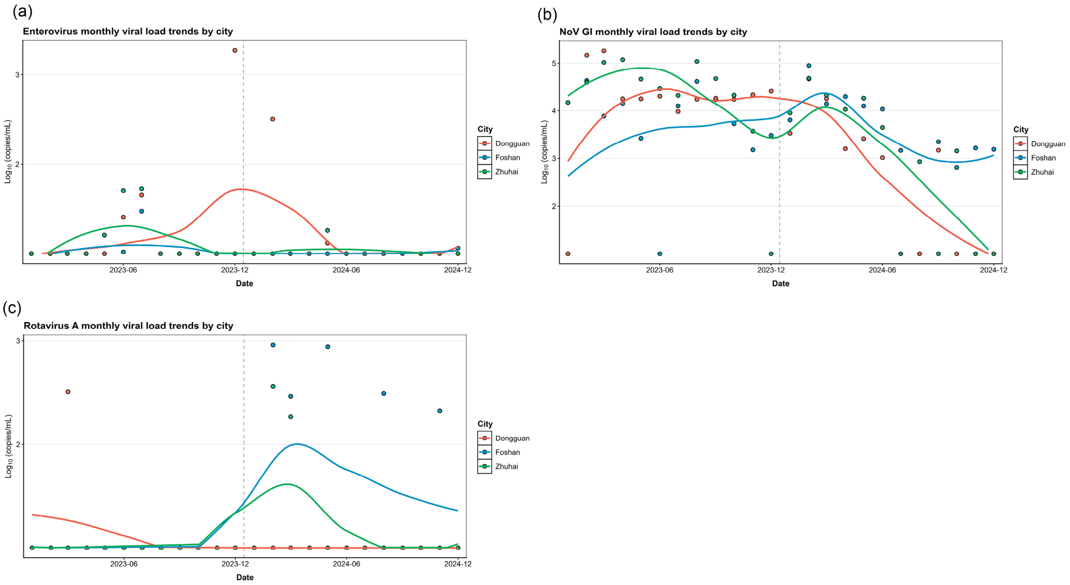

Figure S1. Monthly temporal trends of Enterovirus, Norovirus GI, and Sapovirus viral loads in wastewater samples across three cities. (a) Enterovirus, (b) NoV GI, and (c) Sapovirus.

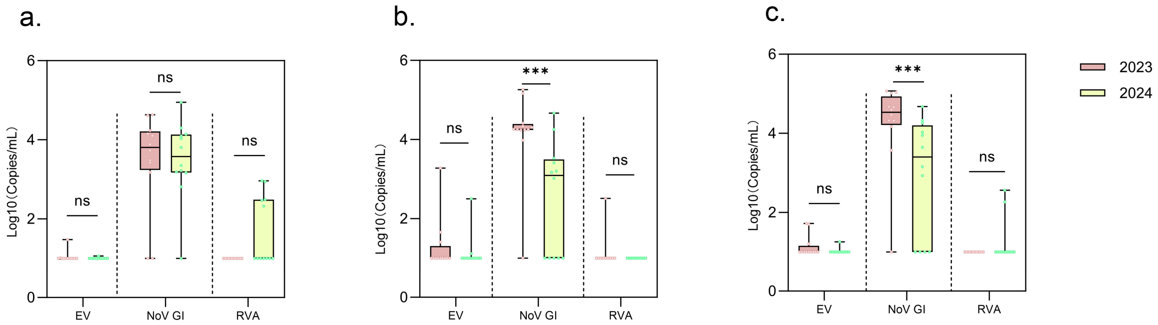

Figure S2. Temporal comparison of Rotavirus A, Enterovirus, and Norovirus GI viral loads in wastewater samples from three cities in Guangdong, China (2023 vs. 2024). (a) Foshan, (b) Dongguan, and (c) Zhuaihai.

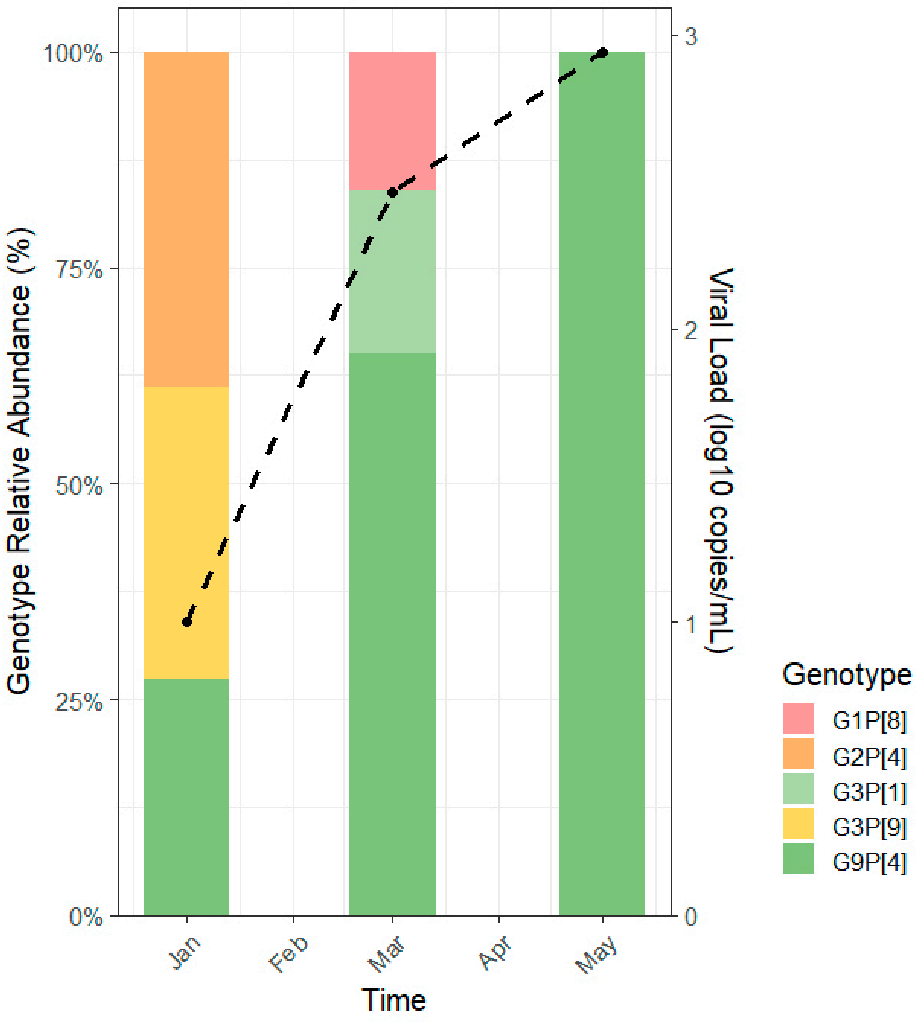

Figure S3. Temporal dynamics of viral genotypes and viral load. Relative abundance of RVA genotypes over time. The stacked bars show the proportion (left y-axis) of different genotypes (indicated by color). The black line tracks the corresponding viral load (right y-axis, $\log_{10}$ copies/mL). The results reveal shifts in genotype composition and viral load throughout the sampling period.
